# Syringaresinol Alleviates Oxaliplatin-Induced Neuropathic Pain Symptoms by Inhibiting the Inflammatory Responses of Spinal Microglia

**DOI:** 10.3390/molecules27238138

**Published:** 2022-11-23

**Authors:** Ji Hwan Lee, Jong Hee Choi, Jaihwan Kim, Tai Wan Kim, Ji-Young Kim, Geehoon Chung, Ik-Hyun Cho, Dae Sik Jang, Sun Kwang Kim

**Affiliations:** 1 Department of Physiology, College of Korean Medicine, Kyung Hee University, Seoul 02447, Republic of Korea; 2 Department of Convergence Medical Science, College of Korean Medicine, Kyung Hee University, Seoul 02447, Republic of Korea; 3 Department of East-West Medicine, Graduate School, Kyung Hee University, Seoul 02447, Republic of Korea; 4 Department of Korean Medicine, Graduate School, Kyung Hee University, Seoul 02447, Republic of Korea; 5 Department of Biomedical and Pharmaceutical Sciences, Graduate School, Kyung Hee University, Seoul 02447, Republic of Korea

**Keywords:** inflammation, microglia, neuropathic pain, oxaliplatin, phytochemical, syrinagresinol

## Abstract

Oxaliplatin-induced peripheral neuropathy (OIPN) is a serious side effect that impairs the quality of life of patients treated with the chemotherapeutic agent, oxaliplatin. The underlying pathophysiology of OIPN remains unclear, and there are no effective therapeutics. This study aimed to investigate the causal relationship between spinal microglial activation and OIPN and explore the analgesic effects of syringaresinol, a phytochemical from the bark of Cinnamomum cassia, on OIPN symptoms. The causality between microglial activation and OIPN was investigated by assessing cold and mechanical allodynia in mice after intrathecal injection of the serum supernatant from a BV-2 microglial cell line treated with oxaliplatin. The microglial inflammatory response was measured based on inducible nitric oxide synthase (iNOS), phosphorylated extracellular signal-regulated kinase (p-ERK), and phosphorylated nuclear factor-kappa B (p-NF-κB) expression in the spinal dorsal horn. The effects of syringaresinol were tested using behavioral and immunohistochemical assays. We found that oxaliplatin treatment activated the microglia to increase inflammatory responses, leading to the induction of pain. Syringaresinol treatment significantly ameliorated oxaliplatin-induced pain and suppressed microglial expression of inflammatory signaling molecules. Thus, we concluded that the analgesic effects of syringaresinol on OIPN were achieved via the modulation of spinal microglial inflammatory responses.

## 1. Introduction

Oxaliplatin is a widely used first-line chemotherapeutic drug for the treatment of advanced metastatic colorectal cancer [1]. Despite its potency in decreasing tumor size, the use of oxaliplatin can be limited because of peripheral neuropathy, a serious side effect that outbreaks 24–48 h after its infusion. Oxaliplatin-induced peripheral neuropathy (OIPN) occurs in up to 90% of treated patients and is often accompanied by symptoms such as sensory loss, paresthesia, and dysesthesia [2,3]. These OIPN symptoms are characterized by a glove-and-stocking distribution and can be triggered and worsened by exposure to causal sensations, such as cold and mechanical stimuli [4]. Conventional painkiller drugs hardly show sufficient efficacy against these neuropathic allodynia symptoms. Although several drugs (e.g., gabapentin and duloxetine) are prescribed to ease these allodynia symptoms in the clinic [5], side effects such as nausea, somnolence, and vomiting limit their use [6].

Previous studies have investigated the various underlying mechanisms of OPIN. Recently, the role of glial activation in OIPN has been revealed, and microglia have been suggested as therapeutic targets for drug development [7,8,9]. Microglia are resident immune cells that comprise large proportions of the central nervous system. In neuropathic pain conditions, microglia are activated to mediate several inflammatory responses [10]. Once microglial activation occurs, the production of various pro-inflammatory enzymes and proteins, such as inducible nitric oxide synthase (iNOS), nuclear factor-kappa B (NF-κB), and mitogen-activated protein kinase (MAPK) including extracellular signal-regulated kinase (ERK), is promoted [11,12]. These inflammatory mediators play a role in the processing and induction of neuropathic pain [13,14] through the synthesis of nitric oxide (NO), which is involved in pain generation [15], prostaglandin E2 (PGE2) [16,17], and other pro-inflammatory cytokines [18,19]. Inflammatory responses involved in pain symptoms of OIPN are also concomitant with spinal glial activation [9,20,21]. Pro-inflammatory cytokines, such as interleukins (IL-1β and IL-6) and tumor necrosis factor-α (TNF-α) [22,23] stimulate neurons or glial cells in the spinal cord, resulting in neuronal hyperexcitation in OIPN [21,24,25].

**Figure 1:**
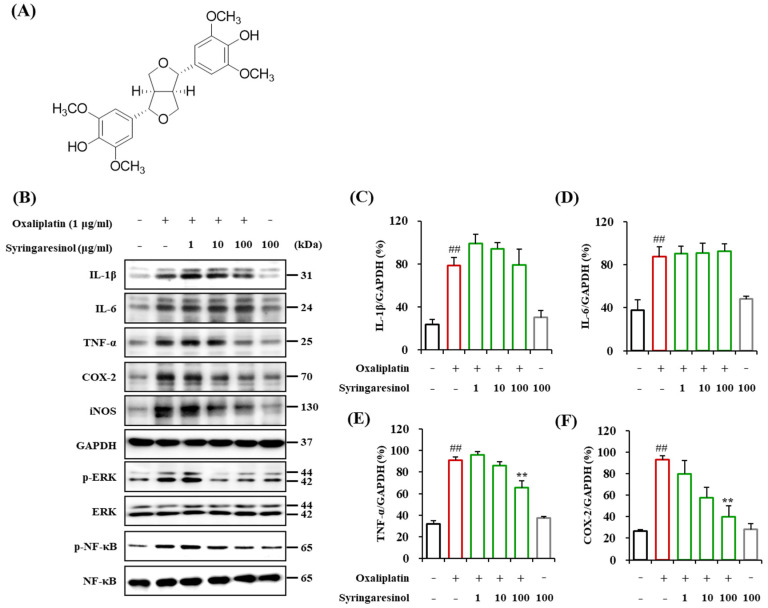

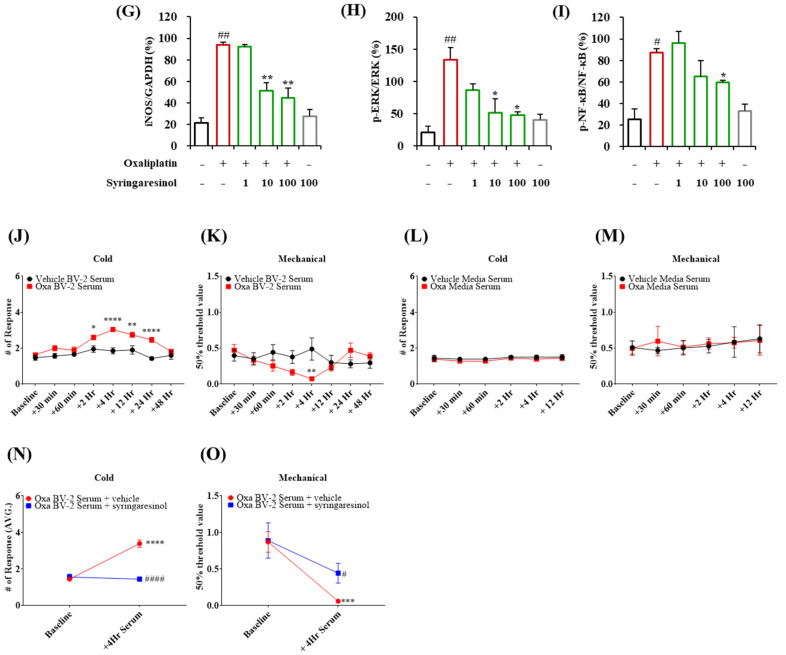
Oxaliplatin treatment activated microglia in BV-2 resulting in the increased expression of inflammatory mediators that can induce pain behavior when intrathecally injected, which was suppressed by syringaresinol treatment. (**A**) The chemical structure of syringaresinol. (**B**–**I**) Western blot analysis of representative proinflammatory cytokines (IL-1β, IL-6, and TNF-α), enzymes (COX-2 and iNOS), and signaling molecules (p-ERK and p-NF-kB) in the BV-2 cell line. Ratio of protein expressions relative to GAPDH or total forms (ERK and NF-kB) expression. Syringaresinol treatment significantly suppressed the oxaliplatin-induced upregulation of inflammatory mediators. # *p* < 0.05 and ## *p* < 0.01 vs. vehicle treated BV-2 cells; * *p* < 0.05 and ** *p* <0.01 vs. Oxa treated BV-2 cells with one-way ANOVA with a Tukey post hoc test to compare multiple groups. (**J**–**M**) A single intrathecal injection of Oxa BV-2 serum elicited cold (**J**) and mechanical allodynia (**K**) in naïve mice. Vehicle BV-2 Serum: *n* = 5, Oxa BV-2 Serum: *n* = 9. * *p* < 0.05, ** *p* < 0.01, *** *p* < 0.001, **** *p* < 0.0001 vs. Vehicle BV-2 Serum with two-way ANOVA followed by Sidak’s post-test for multiple comparisons. (**N**) Intrathecal injection of syringaresinol completely canceled out cold allodynia induced by Oxa BV-2 serum. (**O**) Mechanical allodynia induced by Oxa BV-2 serum was significantly ameliorated by syringaresinol treatment. Oxa BV-2 Serum + vehicle: *n* = 6, Oxa BV-2 Serum + syringaresinol: *n* = 6. *** *p* < 0.001, **** *p* < 0.0001 vs. Baseline and # *p* < 0.05, #### *p* < 0.0001 vs. Oxa BV-2 Serum + vehicle with two-way ANOVA followed by Sidak’s post-test for multiple comparisons.

Syringaresinol is a furofuran type lignan (Figure 1A) found in various medicinal herbs, such as *Panax ginseng* berries, *Magnolia thailandica*, and *Cinnamomum cassia* [26,27,28]. Plant-derived syringaresinol has antibacterial, insecticidal [29,30], anti-inflammatory, and antioxidant effects [31,32]. Syringaresinol directly affects microglia and macrophages to modulate the production of NO and related proteins, such as iNOS, and downregulates inflammatory signaling via the MAPKs pathway [22,33,34,35]. In our previous study, we investigated the analgesic effects of syringaresinol isolated from the bark of *Cinnamomum cassia* (Cinnamomi Cortex) on neuropathic pain induced by paclitaxel [36]. We also found that Cinnamomi Cortex has analgesic efficacy against OIPN [21]. However, the analgesic effect of syringaresinol on OIPN has not been reported.

In this study, we first investigated the involvement of inflammatory responses in spinal microglia in the development of neuropathic pain symptoms in OIPN. Subsequently, we tested whether syringaresinol could suppress oxaliplatin-induced inflammatory responses of activated microglia to exert an analgesic effect.

## 2. Results

### 2.1. Involvement of Microglial Inflammatory Signaling in the Pain Symptoms of OIPN

To investigate whether oxaliplatin could induce inflammatory responses in microglia, the levels of protein expressions of representative inflammatory cytokines (IL-1β, IL-6, and TNF-α), enzymes (cyclooxygenase-2(COX-2) and iNOS), and signaling molecules (p-ERK and p-NF-kB) following oxaliplatin treatment were measured in the BV-2 microglial cell line (Figure 1B–I). Oxaliplatin treatment (3 h, 1 μg/mL) to the BV-2 cell line induced a significant increase in the expressions of IL-1β (Figure 1B,C), IL-6 (Figure 1B,D), TNF-α (Figure 1B,E), COX-2 (Figure 1B,F), iNOS (Figure 1B,G), p-ERK (Figure 1B,H), and p-NF-kB (Figure 1B,I). We tested the involvement of oxaliplatin-induced microglial changes in the manifestation of pain symptoms. BV-2 cells were treated with oxaliplatin (1 μg/mL) for 3 h, and the supernatant was collected from the culture media. We found that a single intrathecal injection of the supernatant serum (Oxa BV-2 serum, 10 μL) induced cold and mechanical allodynia in naïve mice (Figure 1J,K). For cold allodynia, the first significant behavioral change was observed at 2 h after intrathecal injection, and hypersensitivity was reversed at 48 h after injection (Figure 1J). For mechanical allodynia, the 50% paw withdrawal threshold value was significantly reduced at 4 h after injection and was reversed to the control level at 12 h after injection (Figure 1K). Control group animals treated with the supernatant collected from vehicle (1% dimethyl sulfoxide (DMSO)/DPBS)-treated BV-2 cells (vehicle BV-2 serum) did not show behavioral changes. Other control animals injected with the supernatant from media treated with vehicle (vehicle media serum) or media treated with oxaliplatin (Oxa media serum) also showed no behavioral changes (Figure 1L,M). These data indicate that oxaliplatin treatment can cause microglial activation and that microglial changes are directly involved in the symptoms of tactile hypersensitivity observed in OIPN.

### 2.2. Suppression of Oxaliplatin-Induced Inflammatory Signaling in Cultured Microglia by Syringaresinol Treatment

We investigated whether syringaresinol could suppress oxaliplatin-induced changes in BV-2 microglia. Oxaliplatin-induced changes in the levels of protein expressions of representative proinflammatory cytokines (IL-1β, IL-6, and TNF-α), enzymes (COX-2 and iNOS), and signaling molecules (p-ERK and p-NF-kB) were quantified in the BV-2 cells pretreated with syringaresinol (Figure 1B–I). We found that syringaresinol inhibited oxaliplatin-induced increase in TNF-α (Figure 1B,E), COX-2 (Figure 1B,F), iNOS (Figure 1B,G), p-ERK (Figure 1B,H), and p-NF-κB expression (Figure 1B,I).

We then tested whether syringaresinol could suppress the hypersensitive behaviors induced by intrathecal injection of the supernatant from BV-2 cells pretreated with oxaliplatin (Oxa BV-2 serum). Mice were treated with Oxa BV-2 serum, and intrathecal injection of syringaresinol was followed at 4 h after serum injection. Syringaresinol treatment successfully ameliorated serum-induced cold and mechanical allodynia. Mice injected with syringaresinol showed significantly fewer behavioral responses to cold stimuli (Figure 1N) and a significantly higher paw withdrawal threshold against mechanical stimuli (Figure 1O) compared to mice treated with the vehicle. We found that syringaresinol treatment completely reversed serum-induced cold allodynia, as the behavioral responses were comparable to baseline levels in mice treated with syringaresinol. Syringaresinol also significantly attenuated the serum-induced mechanical allodynia.

### 2.3. Analgesic Effects of Syringaresinol Treatment on Symptoms of OIPN

**Figure 2:**
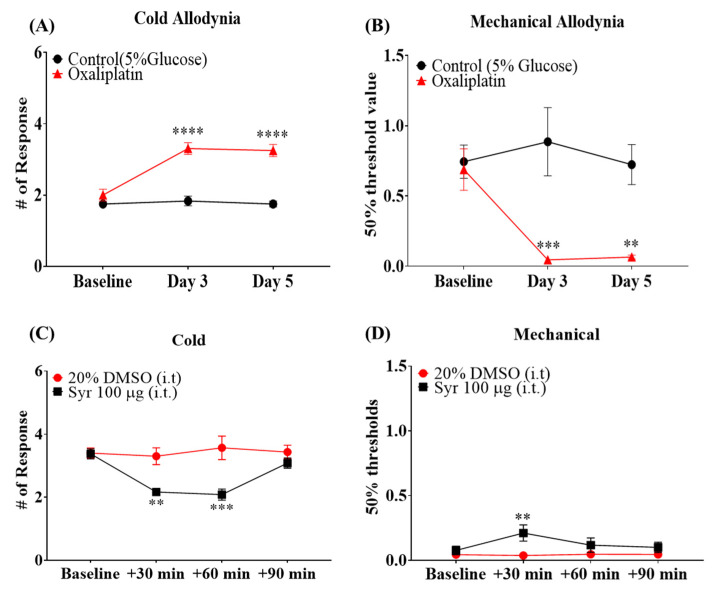
A single injection of oxaliplatin induced cold and mechanical allodynia in mice, which was ameliorated by syringaresinol treatment. (**A**) A single injection of oxaliplatin induced cold allodynia, shown by the increased behavioral response to acetone drop. (**B**) A single injection of oxaliplatin induced mechanical allodynia, shown by decreased paw withdrawal threshold against von Frey filaments. Control: *n* = 6, Oxaliplatin: *n* = 6. ** *p* < 0.01, *** *p* < 0.001, **** *p* < 0.0001 vs. Control with two-way ANOVA followed by Sidak’s post-test for multiple comparisons. (**C**) A single intrathecal injection of syringaresinol attenuated oxaliplatin-induced cold allodynia. (**D**) Single intrathecal injection of syringaresinol attenuated oxaliplatin-induced mechanical allodynia. 20% DMSO: *n* = 5, Syr 100 μg: *n* = 4. ** *p* < 0.01, *** *p* < 0.001, vs. 20% DMSO with two-way ANOVA followed by Sidak’s post-test for multiple comparisons.

A single injection of oxaliplatin (i.p., 6 mg/kg) in mice successfully induced symptoms of OIPN, as shown by changes in behavioral responses to cold or mechanical stimuli. The animals subjected to oxaliplatin injection showed a significant increase in behavioral responses against acetone drop on days 3 and 5 following the injection (Figure 2A), indicating cold allodynia. The animals also showed a significant reduction in the 50% paw withdrawal threshold on days 3 and 5 (Figure 2B), indicating mechanical allodynia. The control group treated with the vehicle (5% glucose solution) did not show a significant change.

To investigate the analgesic effect of syringaresinol on OIPN, syringaresinol was administered to the spinal cord of mice 3 days after the pretreatment of oxaliplatin. Syringaresinol treatment successfully ameliorated oxaliplatin-induced cold and mechanical allodynia. A significant anti-allodynic effect was observed 30 min after injection (Figure 2C,D) and could be further observed at 60 min for cold allodynia (Figure 2C).

### 2.4. Suppression of Oxaliplatin-Induced Spinal Microglial Activation by Intrathecal Injection of Syringaresinol

**Figure 3:**
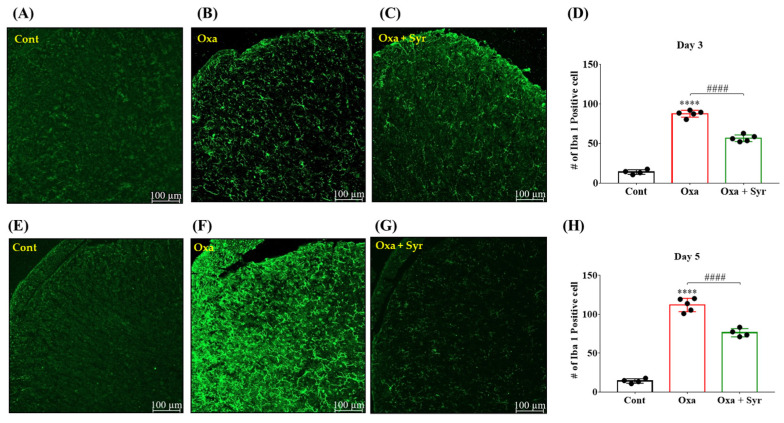
Syringaresinol treatment attenuated oxaliplatin-induced spinal microglia activation. (**A**–**D**) Single intrathecal injection of syringaresinol decreased the number of Iba 1 positive cells at Day3. Representative image of each group; (**A**) Control, (**B**) Oxaliplatin-treated, and (**C**) Oxaliplatin and syringaresinol-treated. (**D**) Total counted number from all the groups. Prominent increase was observed in the oxaliplatin-treated group compared to that in the control. Syringaresinol treatment significantly reduced the oxaliplatin-induced increase of Iba 1 positive cells. (**E**–**H**) Multiple intrathecal injection of syringaresinol lowered the number of Iba 1 positive cells at Day 5. Representative image of each group; (**E**) Control, (**F**) Oxaliplatin-treated, and (**G**) Oxaliplatin and syringaresinol-treated. Counting results were summarized in the graph (**H**). For the data from one mouse, six spinal cord slices were counted and averaged. For Day 3, Cont: *n* = 4, Oxa: *n* = 5, and Oxa + Syr: *n* = 5. For Day 5, Cont: *n* = 4, Oxa: *n* = 5, and Oxa + Syr: *n* = 4. **** *p* < 0.0001 vs. Cont, #### *p* < 0.0001 vs. Oxa with one-way ANOVA followed by Tukey’s post-test for multiple comparisons.

Spinal glial activation has been reported in various pain models, including OIPN [10,21,37,38]. To investigate microglial activation induced by oxaliplatin, the spinal cord was harvested from mice on either day 3 or day 5 following oxaliplatin injection. In addition, we investigated the effect of in vivo treatment with syringaresinol on oxaliplatin-induced spinal microglial activation using spinal cord slices harvested 30 min after intrathecal injection of syringaresinol. Immunohistochemistry was performed using Iba 1 to identify the spinal microglia. The number of Iba 1 positive cells in layers I and II of the dorsal horn laminae was significantly higher in mice injected with oxaliplatin than in the control group. The number of Iba 1 positive cells was significantly decreased by the intrathecal injection of syringaresinol. The oxaliplatin-induced increase and syringaresinol-induced decrease in Iba 1 positive cells were observed both at day 3 (Figure 3A–D) and day 5 (Figure 3E–H).

### 2.5. Suppression of Oxaliplatin-Induced Inflammatory Signaling in the Spinal Microglia by Intrathecal Injection of Syringaresinol

**Figure 4:**
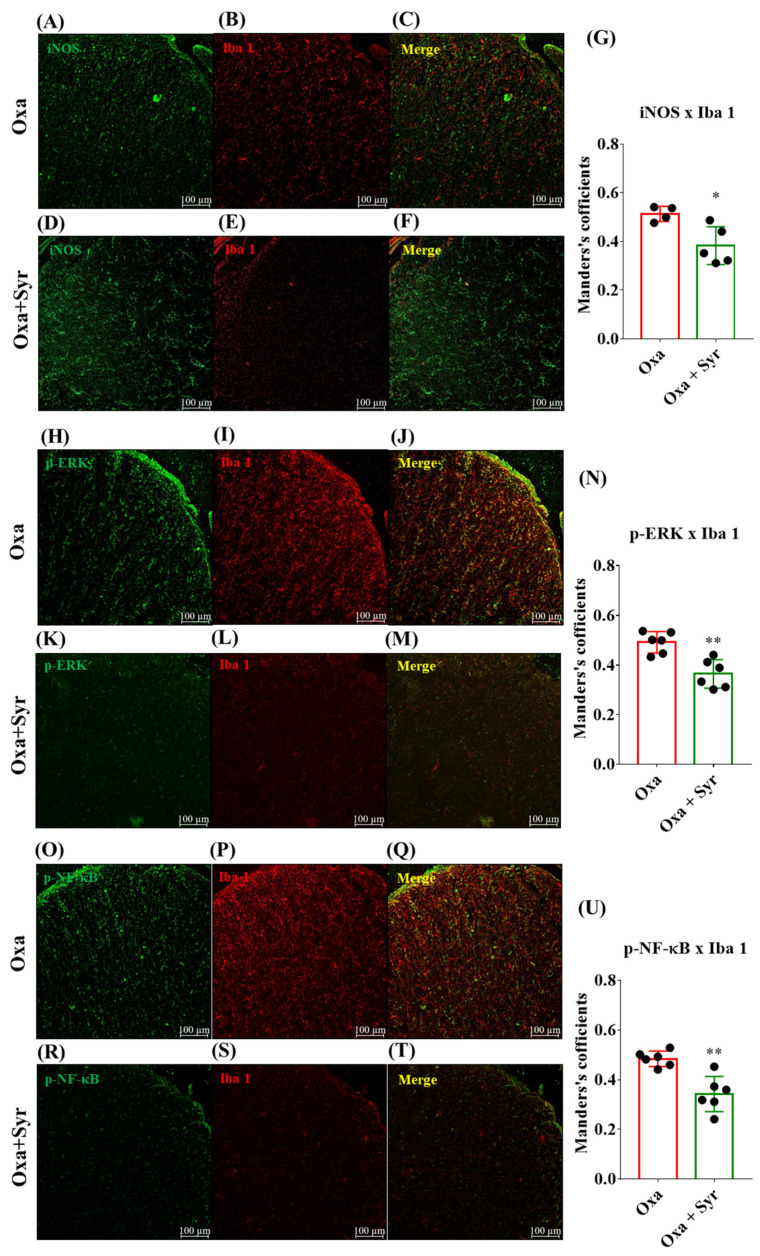
A single injection of syringaresinol downregulates co-localization of Iba 1 and iNOS, p-ERK, or p-NF-kB at the spinal dorsal horn. (**A**–**G**) Representative images of the spinal dorsal horn and a summarized graph showing the colocalizaion of Iba 1 and iNOS; (**A**,**D**) iNOS, (**B**,**E**) Iba 1, and (**C**,**F**) merged image. (**G**) Comparison between co-localization. Syringaresinol treatment significantly reduced the co-localization of Iba 1 and iNOS in oxaliplatin-injected animals. (**H**–**N**) Representative images of -the spinal dorsal horn and a summarized graph showing the co-localization of Iba 1 and p-ERK. (**H**,**K**) p-ERK, (**I**,**L**) Iba 1, and (**J**,**M**) merged image. (**N**) Syringaresinol treatment significantly reduced the co-localization of Iba 1 and p-ERK. (**O**–**Q**) Representative image of the spinal dorsal horn and a summarized graph showing the co-localization of Iba 1 and p-NF-κB. (**O**,**R**) p-NF-κB, (**P**,**S**) Iba 1, and (**Q**,**T**) merged image. (**U**) Syringaresinol treatment significantly reduced the co-localization of Iba 1 and p-NF-κB. For iNOS, Oxa: *n* = 4, and Oxa + Syr: *n* = 5. For, p-ERK, Oxa: *n* = 6, Oxa + Syr: *n* = 6. For p-NF-κB, Oxa: *n* = 6, Oxa + Syr: *n* = 6. * *p* < 0.05, ** *p* < 0.01 by unpaired t-test.

To confirm the effect of syringaresinol on microglial inflammatory signaling in vivo, we performed immunostaining experiments using spinal cord slices obtained from OIPN mice. Antibodies against iNOS, p-ERK, and p-NF-κB were used, as we already confirmed that these inflammatory mediators increased concomitantly with oxaliplatin-induced microglial activation. We quantified the colocalization of these markers with Iba 1, a widely used marker for microglial detection, and measured the effect of a single intrathecal injection of syringaresinol. Compared to the control mice that were treated with 20% DMSO solution as a vehicle, syringaresinol-treated mice showed significantly lower levels of colocalization between Iba1 and either of the tested markers for iNOS (Figure 4A–G), p-ERK (Figure 4H–N), and p-NF-κB (Figure 4O–U).

## 3. Materials and Methods

### 3.1. Experimental Animals

Six-week-old C57BL/6J mice were purchased from DBL (Chungbuk, Korea). All experimental animals were housed with four to five animals per cage. The animal facility has been maintained with a temperature and humidity of 23 ± 2 °C and 65% ± 5%, respectively. Animals were supplied with food and water ad libitum and underwent a fixed 12 h light/dark cycle. All experimental protocols were approved by the Kyung Hee University Animal Care and Use Committee (KHUASP(SE)-20-147) and performed according to the guidelines of the International Association for the Study of pain (IASP) [39]. A total of 119 animals were used during the study.

### 3.2. Oxaliplatin Preparation and Administration

Oxaliplatin (Sigma Aldrich, St. Louis, MO, USA) was dissolved in a 5% glucose solution at a concentration of 2 mg/mL, in accordance with previous studies [20,21]. Oxaliplatin was intraperitoneally (i.p.) injected into the animals at a dose of 6 mg/kg. For the control group, the same amount of 5% glucose solution was administered (i.p.). To assess whether oxaliplatin administration induced symptoms of allodynia in mice, behavioral tests were conducted before (baseline) and 3 (day 3) and 5 (day 5) days after the injection. For treatment of oxaliplatin into the BV-2 cell line, oxaliplatin was dissolved with 1% DMSO/DPBS solution.

### 3.3. Behavior Tests

Cold allodynia and mechanical allodynia were measured using the acetone drop and von Frey filament tests, respectively. For acclimation, animals were placed on a metal mesh floor and caged in an inverted clear plastic cage (12 × 8 × 6 cm) for 30 min before the behavioral test.

To assess the behavioral responses to cold stimuli, an acetone drop (10 μL) was applied to the mid-plantar surface of the hind paws. The behavioral responses (flicking and licking) to acetone drops were observed and counted for 15 s. The average number of hind paw responses after the acetone drop was calculated after six repetitive tests (three times per paw).

A series of von Frey filaments (log unit: 2.36, 2.44, 2.83, 3.22, 3.61, 3.84, 4.08, and 4.31; equivalent bending forces of 0.02, 0.04, 0.07, 0.16, 0.4, 0.6, 1, 1.4, and 2 g, Stoelting, Wood Dale, IL, USA) were applied to the hind paws mid-plantar as mechanical stimuli. The behavioral responses against the von Frey filaments were converted into a 50% withdrawal threshold value using the up-and-down method [40,41]. For all behavioral tests, the data from both hind paws were averaged and represented.

### 3.4. Syringaresinol Preparation and Administration

Syringaresinol was obtained as previously described [36]. In brief, the dried *Cinnamomum cassia* bark (6.0 kg) was extracted with hot water (60 L, 100 °C) twice in water bath for 2 h and the solvent was vaporized at 45 °C in vacuo. The hot water extract (150.0 g) was suspended in 1 L of water and consecutively extracted with ethyl acetate (EtOAc) (1.0 L × 3) to obtain EtOAc- (20.8 g) and water-soluble extracts (128.3 g), respectively. The EtOAc-soluble extract (20.8 g) was fractionated over silica gel (70-230 mesh, ϕ 5.0 × 100.0 cm) as the stationary phase with an EtOAc-hexane gradient (from 0/1 to 1/0, *v*/*v*) as the mobile phase to afford 13 fractions (EA1–EA13).

Among the sorted fractions, EA13 was fractionated using Sephadex LH-20 CC with a CH_2_Cl_2_-MeOH mixture (1:1, *v*/*v*) to produce 15 subfractions (EA13-1–EA13–15). Syringaresinol (38.6 mg) was obtained from subfraction EA13-2 using flash chromatography with a Redi Sep-C18 cartridge (26 g, MeOH/H_2_O = 4/6 to 5/5, *v*/*v*).

For inoculation of syringaresinol into the BV-2 cell line, syringaresinol was diluted in 0.06% Tween-80 solution at a concentration of 1 mg/mL. Syringaresinol diluted in a 20% DMSO solution (10 mg/mL) was used for intrathecal injection. At day 3 after the oxaliplatin injection, syringaresinol was administered via intrathecal route.

### 3.5. BV-2 Cell Culture

BV-2 cell line (a type of microglial cell derived from C57/BL6 murine) was generously provided from Prof. Sung Joong Lee (Department of Oral Physiology, School of Dentistry, Seoul National University, Seoul, Korea). Using Dulbecco’s modified Eagle’s medium (DMEM; Thermo Fisher Scientific, Bothell, WA, USA) culture media with mixture of 10% fetal bovine serum (FBS; Thermo Fisher Scientific) and 1% penicillin (Thermo Fisher Scientific), the BV-2 cell line was cultured in an incubator (5% CO_2,_ 37 °C).

For cell viability assay, BV2 cells were seed into 96-well plates at a density of 1 × 10^5^ cells/mL and treated with syringaresinol (0–200 μg/mL) for 24 h at 37 °C. Following incubation, 0.5 mg/mL MTT (3-(4,5-dimeth-ylthiazol-2-yl)-2,5-diphenyltetrazolium bromide; Sigma-Aldrich) solution were added and incubation was continued for a further 2 h at 37 °C, and then supernatant was removed and dissolved in DMSO. The absorbance at 570 nm was measured by a microplate reader (VersaMax; Molecular devices, San Jose, CA, USA) and the results were expressed as the mean percentage of absorbance based on the non-treated cells. The concentrations of the syringaresinol treatment in the main experiments were determined based on the results of the MTT assay (Supplementary Figure S1A).

To induce microglial inflammation responses, the BV-2 cells were incubated with oxaliplatin (1 μg/mL) for 3 h for stimulation. Syringaresinol was treated 1 h prior to oxaliplatin stimulation. For Western blotting, the BV-2 cells were cultured in a 6-well plate at a density of 5 × 10^5^ cells/mL. For in vivo study, BV-2 cells were cultured 2 × 10^6^ cells/mL in a 100 φ dish and the supernatant was harvested after syringaresinol or oxaliplatin treatment.

### 3.6. Western Blotting

To separate the protein from the BV-2 cells, 50 µL protein lysis buffer (pH 7.9, with 1.5 mM MgCl_2_, 10 mM KCl) was added and incubated for 1 h at 4 °C. The separated protein was quantified by Bradford assay (Bio-Rad Laboratories, Inc., Hercules, CA, USA) with bovine serum albumin (BSA; Sigma Aldrich), and 20 µg of protein was loaded on sodium dodecyl sulfate-polyacrylamide gel electrophoresis (SDS-PAGE) and transferred to a polyvinylidene difluoride (PVDF) membrane (Gendepot, Katy, TX, USA). The protein-transferred PVDF membrane was blocked with 5% skim milk (BD Biosciences, San Jose, CA, USA) for 1 h and rinsed with Tris-buffered saline and Tween-20 (TBST). Samples were then incubated with primary antibodies [rabbit anti-interleukin-1 beta (IL-1ß), mouse anti-IL-6, rabbit anti-tumor necrosis factor-α (TNF-α) (Cell Signaling Technology, Danvers, MA, USA), mouse anti-cyclooxygenase-2 (COX-2), mouse anti-iNOS (Santa Cruz Biotechnology, Inc., Dallas, TX, USA), rabbit anti-phospho-extracellular signal-regulated kinase 1/2 (p-ERK), rabbit anti-ERK, rabbit anti-phospho-nuclear factor kappa B (p-NF-κB), rabbit anti-NF-κB, and rabbit anti-glyceraldehyde 3-phosphate dehydrogenase (GAPDH) (Cell Signaling Technology)] diluted in 3% BSA (1: 1000) overnight at 4 °C. After rinsing with TBST (10 min, thrice), the samples were incubated with secondary antibodies (Vector Laboratories Inc., Newark, CA, USA) for 1 h at room temperature. The expression of the bands was observed using an ECL system (BioD, Gwangmyeong-si, Gyeonggi-do, Korea). Protein quantification was performed using a ChemiDoc XRS+ system (Bio-Rad Laboratories, Inc.).

### 3.7. Immunohistochemistry

Animals were deeply anesthetized with isoflurane inhalation (5% in 1:1 = N_2_O:O_2_, *v*/*v*) and perfused with 0.1 M phosphate-buffered saline (PBS) followed by 4% paraformaldehyde (PFA) (BBC Biochemical, McKinney, TX, USA). The spinal lumbar segments (L4/5) were exposed by laminectomy and identified by tracing the projected dorsal roots from their respective dorsal root ganglia (DRG). Collected tissues were post-fixed in 4% PFA overnight at 4 °C, and then soaked in 30% sucrose (dissolved in 0.1 M PBS) (Sigma Aldrich) for 48 h at 4 °C. Using an optimal cutting temperature (OCT) compound (Sakura Finetek, Tokyo, Japan), a frozen mold of the spinal cord segment was prepared and cut at a thickness of 20 μm using a cryostat (Microm HM 505N; Thermo Scientific, USA). Sections of the spinal cord were collected, mounted on glass slides (Matsunami, Osaka, Japan), and dried for 3 h. The sliced tissues were rinsed thrice with PBS and incubated for 45 min in 0.2% Triton X-100 in PBS at room temperature (RT). The blocking procedure was conducted using 3% bovine serum albumin (BSA; BOVOGEN Biologics, Australia) diluted in 0.05% PBST solution (30 min, RT). After rinsing the slide glass with PBS, the spinal cord slices were incubated overnight at 4 °C with primary antibodies: rabbit anti-Iba 1 (1:500; Wako, Japan), mouse anti-iNOS (1:100; BD, USA) and mouse anti-Iba 1 (1:1000; Abcam, USA), rabbit anti-p-NF-κB, and p-ERK (1:1000; Abcam, Boston, MA, USA). After rinsing in PBS, the secondary antibodies were embedded at RT in the dark for 2 h with anti-rabbit and anti-mouse immunoglobulin G (IgG) labeled with Alexa Fluor 488 and Alexa Fluor 594 (1:1000; Invitrogen, Waltham, MA, USA). Immunofluorescent images were acquired using a confocal laser microscope (LSM 800, Zeiss, Germany) with a 20 × 0.5 NA objective lens. In the spinal dorsal horn. Quantitative and co-localization analyses of Iba 1 with iNOS-, p-NF-κB-, and p-ERK- positive cells were performed using ImageJ (https://imagej.nih.gov/ij/ accessed on 2 Feburary 2022 National Institutes of Health, Bethesda, MD, USA).

### 3.8. Statistical Analyses

All data are presented as mean ± standard error of the mean (SEM). Statistical analyses were performed using Prism 7.0 (GraphPad Software, La Jolla, CA, USA). One-way analysis of variance (ANOVA) was performed, followed by Tukey’s and Fisher’s least significant difference (LSD) tests for multiple comparisons. Two-way ANOVA was performed, followed by Sidak’s post hoc test for multiple comparisons. In all cases, a *p*-value of <0.05 was considered statistically significant.

## 4. Discussion

In this study, we found that microglial activation was associated with allodynia symptoms following oxaliplatin treatment. We further demonstrated that syringaresinol treatment ameliorated oxaliplatin-induced cold and mechanical allodynia by suppressing microglial activation. Oxaliplatin-induced microglial activation was accompanied by the upregulation of pro-inflammatory mediators, including iNOS, p-NF-κB, and p-ERK, which could be suppressed by syringaresinol treatment.

Our findings highlight the importance of spinal microglia in the allodynic symptoms of OIPN. Although the mechanisms of OIPN have not been elucidated, accumulating evidence indicates that the activation of spinal microglia mediates the symptoms of oxaliplatin-induced neuropathic pain [9,37,42]. Similar to several other types of neuropathic pain [43,44], spinal glial activation has been observed in the OIPN model, followed by pain behavior [9,42]. In this study, we also confirmed microglial activation following oxaliplatin administration in cultured BV-2 or the spinal cord in vivo. The involvement of microglial activation in pain behavior in OIPN was further investigated using supernatant serum isolated from BV-2 cells treated with oxaliplatin. Naïve animals developed allodynia symptoms after intrathecal injection of the serum.

Microglial activation-induced upregulation of inflammatory signaling mediators is critically related to the development of neuropathic pain. Microglia rapidly turn into an activated form in response to stimuli that threaten physiological homeostasis, such as nerve damage [10]. Activated microglia secrete various neurotoxic mediators, such as nitric oxide (NO), iNOS, COX-2, prostaglandin E2 (PGE2), and pro-inflammatory cytokines, including TNF-α and interleukins (IL-1β, IL-6) [45]. Indeed, direct stimulation of the satellite glial cell culture by oxaliplatin alters the functions of SGC, leading to the release of pro-inflammatory cytokines [46]. Inflammatory mediators released by activated microglia act as gliotransmitters that stimulate neurons or glia to induce long-term changes, leading to pain symptoms. Previous studies have revealed that spinal glial activation participates in the development of pain via long-term potentiation (LTP). Spinal gliogenic LTP induced by high-frequency electrical stimulation can be transferred by implanting cerebrospinal fluid from LTP-induced animals to naïve animals [47], and intrathecal injection of stimulants derived from spinal glial cells to naïve animals elicits pain behavior [48]. We measured the expression levels of iNOS, p-ERK, and p-NF-κB in the microglia following oxaliplatin treatment. We confirmed that a single administration of oxaliplatin could promote the expression of signaling mediators in the cultured BV-2 cell line as well as in the spinal cord of mice, with morphological changes and proliferation of microglia.

The upregulation of iNOS, p-ERK, and p-NF-κB is accompanied by neuroinflammation. Neuroinflammation plays a role in the development of several types of neuropathic pain including chemotherapy-induced peripheral neuropathy [38,49,50]. During the development of neuropathic pain, microglial cells are activated in response to harmful stimuli or nerve damage and induce inflammatory responses. Microglia promote neuroinflammation via NO and iNOS production [34,51], phosphorylation of transcription factors such as NF-κB and MAPKs such as ERKs [52], and the release of signaling mediators including IL-1β, IL-6, TNF-α, PGE2, and brain-derived neurotrophic factor (BDNF). These inflammatory signaling cascades reinforce neuronal excitability and eventually lead to central sensitization [17]. The activation of microglia and an increase in TNF-α and IL-1β levels in the spinal cord have been reported in OIPN [20,21]. These inflammatory events associated with microglial activation, and following changes of astrocytes and neurons, are critically involved in the onset of chronic pain symptoms. Considering that chronic neuropathic pain is hardly reversed once onset, interfering with the development of symptoms at an early stage can be an important strategy for disease prevention. In this regard, we sought to test whether the drug candidate can inhibit oxaliplatin-induced microglial activation to control pain symptoms.

We investigated the effects of syringaresinol, a lignan isolated from *Cinnamomum cassia* [53,54], on microglial changes induced by oxaliplatin. Syringaresinol has anti-inflammatory [31] and antioxidant effects [55] similar to other phenolic compounds in natural products [56]. The pain modulatory effect can also be induced via the inhibition of pro-inflammatory cytokine release [57]. According to previous studies, syringaresinol treatment attenuated NO synthesis, NF-κB signaling, and MAPKs signaling in LPS-stimulated BV-2 or RAW 264.7 [22,51,58] to induce anti-inflammatory effects. Consistent with these results, our experimental data demonstrated that oxaliplatin-induced upregulation of iNOS, p-ERK, and p-NF-κB was attenuated by syringaresinol treatment. The activation of microglia was also inhibited. Consequently, syringaresinol treatment successfully inhibited pain behaviors induced by either the direct administration of oxaliplatin or intrathecal injection of serum isolated from BV-2 cells pretreated with oxaliplatin.

## 5. Conclusions

In conclusion, we report the critical role of microglial activation in the development of pain in OIPN and suggest syringaresinol as a novel therapeutic agent. OIPN is a major obstacle that hinders the continued use of chemotherapy in cancer patients, and there are currently no identified treatments that completely address the serious side effects. Our study contributes to the establishment of therapeutic strategies by revealing that microglial activation underlies OIPN symptoms. Furthermore, we propose syringaresinol as a substance that could be used to develop novel therapeutics. Given that microglial activation and neuroinflammation are also involved in numerous other neurological diseases, syringaresinol could be used not only for OIPN, but also for various other disorders.

## 6. Patents

J.H.L., J.H.C., I.H.C., D.S.J. and S.K.K. hold patents related to the contents of this article (registration #:10-2368413 in Korea).

## Data Availability

The data for this study are available from the corresponding authors upon reasonable request.

## Supplementary Materials

The following supporting information can be downloaded at: https://www.mdpi.com/article/10.3390/molecules27238138/s1, Figure S1: MTT assay to evaluate the cell viability of BV-2 cells.

## Sample Availability

Samples of the compounds are not available from the authors.
