# Supplementary material for: Syringaresinol Alleviates Oxaliplatin-Induced Neuropathic Pain Symptoms by Inhibiting the Inflammatory Responses of Spinal Microglia
Source: Molecules. 2022 Nov 23;27(23):8138. doi: 10.3390/molecules27238138 (PMC9736412; doi:10.3390/molecules27238138)
Supplement: Supplementary file 1 [file molecules-27-08138-s001.zip › molecules-2017314-supplementary.pdf]

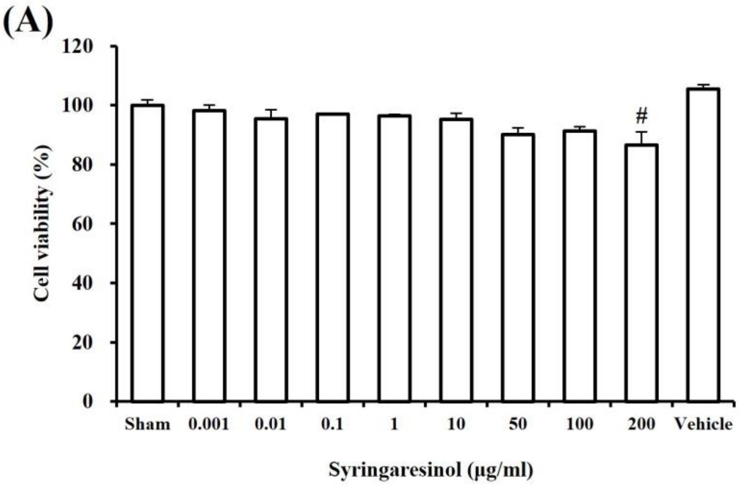

Supplementary Figure S1. MTT assay to evaluate the cell viability of BV-2 cells. (A) MTT assay showed that treatment of syringaresinol with a concentration of 200 μg/mL could be cytotoxic. # $p < 0.05$ compared to sham control.
